# Neurological and cognitive impairment in long‐term COVID‐19: clinical characterization in a longitudinal study and correlation with neuroimaging and biomarkers

**DOI:** 10.1002/alz.083676

**Published:** 2025-01-09

**Authors:** José Wagner Leonel Tavares Júnior, Manoel Alves Sobreira Neto, Pedro Braga‐Neto

**Affiliations:** ^1^ Federal Universtity of Ceará, Fortaleza, CEARÁ Brazil; ^2^ Hospital Universitário Walter Cantídio, Fortaleza, CEARÁ Brazil; ^3^ UNIVERSIDADE FEDERAL DO CEARÁ, Fortaleza, CEARÁ Brazil

## Abstract

**Background:**

COVID‐19 can course with persistent symptoms after infection in a condition called long Covid (NATH, 2020). In this context, cognitive complaints, sleep disorders, headache, smell disorders, in addition to anxiety and depression are common (DELGADO‐ALONSO et al, 2022; ISMAEL et al, 2021.). The present study aims to describe the cognitive manifestations in patients who had a previous COVID‐19 infection and is justified by the need to better explain the pathophysiological mechanisms behind these manifestations.

**Method:**

This is a cross‐sectional view of a prospective longitudinal study carried out on an outpatient basis. Patients have been evaluated on an outpatient basis since March 2023. Patients were evaluated by a neurologist and sleep specialist, with the application of MMSE, GDS/BECK, Pittsburgh, Epworth, Pfeffer and CDR. In addition, a blood sample is collected to analyze APOE and markers of neurodegenerative and inflammatory processes (NfL, AB42, AB40, p‐tau, t‐tau, GFAP, alpha‐synuclein and clusterin). In addition, all patients will undergo cranial MRI, polysomnography and neuropsychological evaluation. The project is approved by the local CEP, all patients have signed an informed consent form.

**Result:**

So far, 93 patients have been evaluated, with 79% female. 1.1% had <4 years of schooling, 4.5% between 4‐8 years and 94.3% > 8 years of schooling. The average duration of symptoms was about 2 years. The average age was 42.8 years. During the illness, only 13.9% were hospitalized, and 10.7% in the ICU. As for the applied batteries, 4.3% of the patients presented alterations in the Pfeffer, and 3.2% presented alterations in MMSE for education. As for post‐Covid symptoms, 96.7% had cognitive complaints, 73.1% sleep disorders, 63.4% anxiety, 35.4% headache, 32.2% depression and 24.7% smell disorders. Results of neuropsychological assessment, biomarkers, cranial MRI and PSNG are pending.

**Conclusion:**

In our study, cognitive manifestations and sleep disturbances were common in the Long Covid phase, even in a population with x months after the acute infection. In addition, cognitive complaints were common even in a sample with a mean age < 50 years, after a mild case of Covid‐19 and with a high level of education.

